# Seafood-Associated Outbreak of *ctx*-Negative *Vibrio mimicus* Causing Cholera-Like Illness, Florida, USA

**DOI:** 10.3201/eid2910.230486

**Published:** 2023-10

**Authors:** Meer T. Alam, Sarah R. Stern, Devin Frison, Katie Taylor, Massimiliano S. Tagliamonte, S. Sakib Nazmus, Taylor Paisie, Nicole B. Hilliard, Riley G. Jones, Nicole M. Iovine, Kartik Cherabuddi, Carla Mavian, Paul Myers, Marco Salemi, Afsar Ali, J. Glenn Morris

**Affiliations:** University of Florida Emerging Pathogens Institute, Gainesville, Florida, USA (M.T. Alam, M.S. Tagliamonte, S.N. Sakib, T. Paisie, C. Mavian, M. Salemi, A. Ali, J.G Morris, Jr.);; University of Florida College of Public Health and Health Professions, Gainesville (M.T. Alam, S.S. Nazmus, A. Ali);; University of Florida College of Medicine, Gainesville (S.R. Stern, K. Taylor, M.S. Tagliamonte, T. Paisie, R.G. Jones, N.M. Iovine, K. Cherabuddi, C. Mavian, M. Salemi, J.G. Morris, Jr.);; Florida Department of Health in Alachua County, Gainesville (D. Frison, P. Myers);; University of Florida Health and Shands Hospital, Gainesville (N.B. Hilliard, N.M. Iovine, K. Cherabuddi)

**Keywords:** Vibrio mimicus, seafood, diarrhea, food safety, bacteria, enteric infections, Florida, United States

## Abstract

*Vibrio mimicus* caused a seafood-associated outbreak in Florida, USA, in which 4 of 6 case-patients were hospitalized; 1 required intensive care for severe diarrhea. Strains were *ctx-*negative but carried genes for other virulence determinants (hemolysin, proteases, and types I–IV and VI secretion systems). Cholera toxin–negative bacterial strains can cause cholera-like disease.

*Vibrio mimicus*, named because of its close metabolic and genetic similarity to *V. cholerae*, is recognized globally as a cause of foodborne and waterborne diarrheal disease (*1*–*4*). Limited data indicate that *V. mimicus* incidence is lower than that reported for *V. parahaemolyticus* and non-O1/non-O139 *V. cholerae* but comparable to that of *V. fluvialis* (*3*,*4*). Although some *V. mimicus* strains produce cholera toxin (CTX) or a cholera-like toxin or have genes from the *ctx* complex, most do not (*1*,*5*). Nonetheless, *V. mimicus* can cause severe, cholera-like illness; the hospitalization rate among case-patients reported in 2014 (the most recent year for which data are available) to the Centers for Disease Control and Prevention is 57% (*3*). We report a seafood-associated outbreak caused by *V. mimicus* in Florida, USA, in which 4 of 6 patients required hospitalization.

## The Study

In June 2019, the Florida Department of Health in Alachua County (DOH-Alachua; Gainesville, FL, USA) received reports of multiple cases of diarrheal illnesses associated with eating at a local seafood restaurant. Six case-patients were subsequently identified who met the case definition of having eaten seafood at the implicated restaurant within a 2-day time window and who experienced acute onset of diarrhea within 96 hours of the reported meal or had a clinical diagnosis of vibriosis. Median case-patient age was 35.5 years; median incubation period was 24 (range 7–32) hours. All 6 case-patients had diarrhea; other signs/symptoms were vomiting (6 case-patients), headache (3 case-patients), and nausea (3 case-patients). Four patients were hospitalized, 3 at the University of Florida Heath Science Center (UFHealth; Gainesville). Among the UFHealth patients, 1 was admitted to the intensive care unit because of a diarrheal purge of 7–8 L. That patient was 39 years of age, had a history of hypertension and type 2 diabetes mellitus, and fully recovered after treatment with volume repletion and 4 days of doxycycline and ceftriaxone.

DOH-Alachua determined that the foods most commonly consumed by case-patients were steamed blue crab (5 case-patients), steamed snow crab (5 case-patients), and steamed shrimp (4 case-patients). Only 1 case-patient reported eating oysters. A joint environmental health assessment by DOH-Alachua, the Florida DOH regional environmental epidemiologist, and the Florida Department of Business and Professional Regulation documented multiple food safety violations (i.e., substantive overall sanitation issues, thawing frozen shrimp overnight at room temperature, returning cooked crabs to crates that previously held live crabs), and a lack of required state-approved employee education.

Fecal samples from the patients hospitalized at UFHealth were initially screened by using a culture-independent diagnostic PCR technique (BioFire FilmArray GI Panel; BioFire Diagnostics, https://www.biofiredx.com), which indicated possible *V. cholerae* and non-*cholerae* *Vibrio* spp. In follow-up studies at the University of Florida Emerging Pathogens Institute, fecal samples were plated on thiosulfate citrate bile-salts sucrose agar after enrichment in alkaline peptone water (pH 8.5) (Appendix). Green colonies, reflecting the lack of sucrose fermentation, characteristic of *V. mimicus*, grew from 2 of 3 samples. We confirmed that the isolates were *V. mimicus* by using convergent PCR primers (*6*); we designated the strains as D461B_US_2019 and E3_US_2019. Isolates were susceptible to doxycycline, azithromycin, ciprofloxacin, and ceftriaxone (Appendix Table 1).

Neither strain carried genes encoding CTX (*ctx*A or *ctx*B) or other accessory genes linked to the CTX phage. However, both strains elicited strong hemolysis/hemolytic activity against sheep blood as measured by standard assays (Appendix Figure 1). Strains also exhibited protease activity, motility, and biofilm formation (Appendix Figures 2, 3). Whole-genome sequencing and bioinformatic analysis detected genes for a variety of possible virulence determinants, including hemolysin, various proteases, and types I–IV and VI secretion systems (Appendix Table 2). Strains carried both a heat stable and heat labile toxin, which showed >98% nucleotide and amino acid identity to similar genes in a *V. mimicus* strain (SCCF01) that was hypervirulent for freshwater catfish in China (*7*). In an earlier genomic study of 2 *ctx*-negative *V. mimicus* strains, Hasan et al. (*1*) reported the presence of genetic elements of the pre-CTX prophage (including *ace* and *zot*, implicated as contributors to diarrhea associated with CTX-negative *V. cholerae* strains [*8*]) and the VSP-II pathogenicity island, neither of which were present in our Florida *V. mimicus* strains. They also noted a gene cluster similar to that of the *V. cholerae* VPI-2 pathogenicity island; we found an identical gene cluster in our strains.

In a phylogenetic analysis based on the core genome (*9*) (Appendix), the 2 Florida strains clustered within a single, well-supported clade consistent with their high similarity, indicating a very recent common origin (Figure). Although the maximum-likelihood tree of available *V. mimicus* sequences shows several independent lineages of strains sampled worldwide with no apparent geographic structure, the closest lineages to the Florida strains were clinical isolates from the United Kingdom, as well as clinical and environmental strains from the United States, albeit with no strong bootstrap support.

**Figure:**
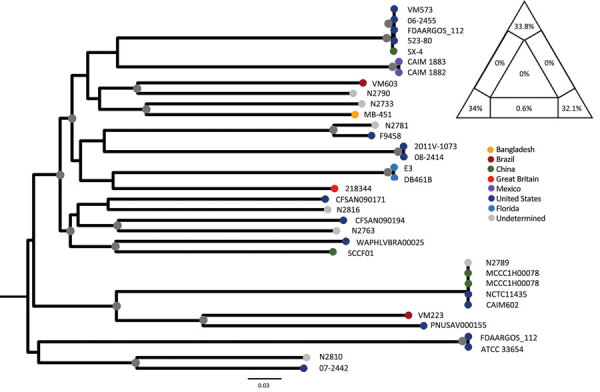
Maximum-likelihood tree of *Vibrio mimicus* strains. The tree was calculated by using 35 sequences; the best evolutionary model was selected by Bayesian information criterion. Nodes marked by a gray circle have bootstrap support (1,000 replicates) >90%. Scale bar represents the number of substitutions per site. The phylogenetic signal of the alignment was determined by likelihood mapping, as shown by the triangle in the top right corner. Likelihood mapping method is based on the analysis of maximum likelihoods for quartets of sequences randomly extracted from the alignment. There are only 3 possible fully resolved tree topologies deriving from 4 sequences (e.g., with 4 sequences named A,B,C, and D, sequence A can cluster with B and C with D; or A with C and D with B; or A with D and B with C); alternatively, if there is not enough information in the sequences, the result will be a star-like topology. The likelihoods of the 3 possible topologies are represented as 1 point in an equilateral triangle, in which each vertex represents 1 of the possible topologies. The triangle is partitioned in 7 regions: the region in the center represents completely unresolved quartets with star-like evolution topology; the 3 regions in the corners represent well-resolved topologies; the regions along the sides represent the situation in which it is difficult to decide between 2 of the 3 possible topologies. Percentages in the triangle reflect the number of quartets assigned to each region. A higher sum of the percentages in the triangle corners (completely resolved quartets) indicates a higher phylogenetic signal contained in the alignment. The alignment signal of >99% therefore shows a strong phylogenetic signal, which enables reliable calculation of the tree.

## Conclusions

*V. mimicus* is present either as a free-living microorganism or in biofilm in the aquatic/estuarine environment; infection is strongly associated with seafood consumption (*4*,*10*,*11*). For this outbreak, we could not identify a single source for the infection, given that case-patients had eaten a variety of seafoods and there were sufficient breakdowns in food safety practices within the kitchen to make cross-contamination of multiple foods highly likely. Although oysters have been implicated as a *V. mimicus* source, only 1 case-patient reported eating oysters, making oysters an unlikely primary source of infection. We also note the risk inherent in placing cooked seafood back into containers in which live seafood has been shipped, a practice linked with previous *V. mimicus* and other *Vibrio* spp. outbreaks (*12*).

Most hospitals and commercial laboratories have now moved to use of culture-independent diagnostic techniques, resulting in a strikingly increased number of cases of *Vibrio*-associated infections reported in the United States (*13*). The outbreak reported here highlights the utility of such techniques for providing a quick, preliminary diagnosis of a possible *Vibrio* infection as well as problems with such systems. Exact isolate speciation was not possible with the system used by UFHealth, and speciation subsequently required careful classical microbiological approaches (with enrichment procedures) to isolate and identify the organism, without which this outbreak caused by a less common *Vibrio* species would almost certainly have been missed.

*V. mimicus* evolved from a common *V. cholerae* ancestor with a prototypic sixth pandemic genomic backbone (*1*). Although most *V. mimicus* strains have not retained the *ctx* genes (the primary virulence factor responsible for the severe diarrhea seen in cholera), the 2 strains isolated in this outbreak carried a wide variety of potential virulence determinants, showing varying degrees of similarity with potential virulence factors reported for other *V. mimicus* strains (*1*,*7*). Common to many other *Vibrio* species (including *V. cholerae*), *V. mimicus* has a superintegron (*1*,*14*), which can serve as a capture system for acquiring DNA from the surrounding environment and may have contributed to the accumulation of virulence genes. We did not see evidence of distinct clinical and environmental lineages (associated with *V. cholerae* and *V. parahaemolyticus*), underscoring the idea that most *V. mimicus* strains, regardless of origin, have the potential for carrying genes capable of causing illness in humans.

Although incidence data for *V. mimicus* are limited, partially because of diagnostic difficulties, *Vibrio* case numbers are clearly increasing with rising surface water temperatures (*13*,*15*,*16*). Thus, diagnostic capabilities for *V. mimicus* and other *Vibrio* species need to be enhanced. There also needs to be recognition that strains that do not carry cholera toxin can cause cholera-like disease and that further work is needed to more clearly identify the pathogenic mechanisms by which such strains cause illness.

Appendix Supplemental information for study of seafood-associated outbreak of *ctx*-negative *Vibrio mimicus* causing cholera-like illness, Florida, USA.
