# Supplementary material for: Seafood-Associated Outbreak of ctx-Negative Vibrio mimicus Causing Cholera-Like Illness, Florida, USA
Source: Emerg Infect Dis. 2023 Oct;29(10):2141–4. doi: 10.3201/eid2910.230486 (PMC10521627; doi:10.3201/eid2910.230486)
Supplement: Appendix — Supplemental information for study of seafood-associated outbreak of ctx-negative Vibrio mimicus causing cholera-like illness, Florida, USA. [file 23-0486-Techapp-s1.pdf]

Article DOI: https://doi.org/10.3201/eid2910.230486

EID cannot ensure accessibility for supplementary materials supplied by authors. Readers who have difficulty accessing supplementary content should contact the authors for assistance.

Seafood-Associated Outbreak of *ctx*-Negative *Vibrio mimicus* Causing Cholera-Like Illness, Florida, USA

Appendix

Initial identification of *V. mimicus* strains. We analyzed three diarrheal stool samples from case-patients at University of Florida Health (UFHealth). Duplicate portions of each stool sample (10 µl) were inoculated in 3 ml alkaline peptone water (APW, pH 8.5) and the culture tube was incubated statically at 37°C for 6–8 h or overnight. Following incubation, a loopful of culture was streaked onto thiosulphate citrate bile salts (TCBS) agar and the plates were incubated overnight at 37°C. After incubation we observed the growth of green colonies in plates with samples from two of the three patients' stool samples, consistent with the presence of a non-sucrose fermenting *Vibrio* spp. We were unable to obtain any colonies (either green or yellow) from the third stool sample.

To confirm the species identification of the TCBS agar-grown green colonies isolated from two of the stool samples a multiplex PCR-based assay was performed as described previously (*1*). Briefly, a single colony obtained from each patient's stool sample was grown in 3-ml L broth and the culture was incubated overnight at 37°C in a shaking incubator (250 rpm). The culture was spun down by centrifugation at 8,000 rpm for 5 minutes; the supernatant was decanted, and the pellet was used for DNA extraction and purification using DNeasy blood and tissue kit (Qiagen, Germantown, MD) following the manufacturer's instructions. PCR primers and conditions to identify *Vibrio* genus and four *Vibrio* species (*V. cholerae* [as a negative control], *V. mimicus*, *V. parahaemolyticus* and *V. vulnificus*) were used as described previously (*1*). The PCR product was subjected to electrophoresis using 0.8% agarose gel to identify an expected band corresponding to *Vibrio* spp. of interest. PCR products were gel purified and sequenced, results consistent with species identification as *V. mimicus*. The strains were

designated as D461B_US_2019 and E3_US_2019, respectively for further analysis as described below.

Hemolysis/hemolytic activity. Qualitative hemolysis assay. A qualitative hemolysin assay was performed as described previously (*2*). Briefly, the two Florida *V. mimicus* strains and two laboratory toxigenic *V. cholerae* O1 strains (E7946 [El Tor Ogawa] and O395 [Classical Ogawa]) were grown statically overnight on LB agar plates at 37°C (strains used in these studies are listed in Appendix Table 2). A single colony grown on LB agar plate was streaked on 5% sheep blood agar plates (Fisher Scientific Co., Waltham, MA) within prelabelled spots drawn on blood agar plate to avoid cross-contamination. The plate was incubated overnight at 37°C in an incubator. Following incubation, the plate was visually observed for hemolytic zone, if any, around each strain. Results are shown in Appendix Figure S1A.

Quantitative hemolysin assay. For a quantitative hemolysin assay, bacterial cell-free culture supernatant [CFS] (extracellular hemolytic activity) and bacterial cytoplasmic extract [CE] (inter cellular hemolytic activity) were determined as follows:

- To obtain bacterial cell-free supernatant, a single colony of each *V. cholerae* strain grown on LB agar was inoculated in 3 ml brain heart infusion (BHI broth); and the culture was incubated overnight at 37°C with a shaking speed of 200 rpm. Following incubation, the culture was transferred into 50 ml Syncase medium supplemented with 3% glycerol (v/v) and the culture was incubated statically at 37°C for 48 hours. The culture was spun down by centrifugation at 12000 rpm for 30 min; the culture supernatant was carefully decanted into a sterile container and ultimately sterilized by membrane (0.45 µm) filtration.
- For preparing bacterial cytoplasmic extract, a single colony of *V. cholerae* strain was inoculated in 3 ml LB broth and the culture was incubated overnight in a shaking incubator with a shaking speed of 200 rpm at 37°C. The culture was then transferred (1:100) in fresh 20 ml LB broth and the culture was grown at 37°C with shaking until it reaches to mid-log phase (value 0.6 at OD_{600}). The culture was spun down by 12000 rpm for 30 min and the pellet was washed 2X with 20 ml fresh LB broth. Supernatant was discarded and the pellet was resuspended in 20 ml LB broth and the culture was chilled on ice for 15 min. Bacterial cells were

then lysed using ultrasonication for a total of 5 min (5 seconds treatment with a 5 second interval). To collect the cytoplasmic extract, ultrasound-treated cells were centrifuged at 12000 rpm for 5 minutes at 4°C. After centrifugation, cells were resuspended in 20 ml LB.

Sheep blood was processed for quantitative hemolytic assay as described previously (*3*). Briefly, 10% sheep blood was washed three times with sterile isotonic saline solution. At the last washing step, erythrocytes were separated by centrifugation at 2000 rpm for 10 min; the supernatants were carefully decanted and a 2% erythrocyte solution (v/v) was prepared by dissolving and mixing erythrocytes into isotonic saline solution. To assess hemolytic activity in CFS and CE (described above), dilution of CFS and CE was made in LB broth as follows: 1:10, 1:20, 1:40 and 1:80. One ml of CFS or CE was added to 1 ml of 2% erythrocyte solution (final concentration of erythrocytes approached to 1%) in 5 ml sterile test tube. The mixture was incubated statically at 37°C for 2 hours followed by incubation in 4°C overnight. Following overnight incubation, 1 ml mixture was spun down by centrifugation at 1000 rpm for 2 min; 200 µl supernatant were transferred to 96-well plate to measure the absorbance at 540 nm. To completely lyse erythrocytes, 1% erythrocytes was treated with 1% Triton-X100 and used as positive control as reported previously (*3*). One ml of erythrocytes solution (1% final concentration) was added to 1 ml of LB broth (free from either CFS or CE) was used as a blank (negative control). The amount of hemolytic toxin necessary to elicit 50% release of hemoglobin or to cause 50% hemolysis was defined as the hemolysin unit (HU_{50}). The relative rate of hemolysis was calculated using the following equation: Hemolysis (%) = 100 – [(Ap-As)/(Ap-An) 100)]; where Ap, As and An are the O.D. value or absorbance of the positive control, test strains and the negative control, respectively. Fifty percent (50%) hemolytic activity was determined based on the 50% hemolysis found in a tube (dilution) compared to the positive control tube (lysis was done by Triton X-100).

Appendix Figure S1B shows that *V. mimicus* strains produced either comparable or higher level of hemolytic activity in their cell free supernatant compared to a positive standard control (completely lysed erythrocytes using Triton X 100). The E3_US_2019 and D461B_US_2019 strains displayed 50% hemolytic activity at a fold dilution of ~29 and ~39, respectively as indicated by dashed lines. The amount of hemolytic toxin required to elicit 50% release of hemoglobin or to cause 50% hemolysis was defined as the hemolysin unit (HU_{50}).

Similarly, Appendix Figure S1C indicated that both *V. mimicus* strains retained similar level of hemolytic activity in their cytoplasmic extract compared to lysed erythrocytes. Both E3_US_2019 and D461B_US_2019 strains displayed 50% hemolytic activity at a fold dilution of ~3.3 as indicated by dashed lines. As in cell free supernatant, the amount of hemolytic toxin (extracted from cytoplasmic extract) required to elicit 50% release of hemoglobin or to cause 50% hemolysis was defined as the hemolysin unit (HU_{50}). Taken together, our data imply that high hemolytic activity seen in cell free supernatant and cytoplasmic extract of *V. mimicus* strains may have contributed to severe diarrheal diseases in patients from which the strains were isolated.

Qualitative protease assay. Qualitative protease activity of *Vibrio* strains was assessed using milk agar plate as described previously (*4*). The milk agar plate containing 4% (w/v) instant non-fat dry milk (autoclave separately before add to agar) was mixed with separately autoclaved brain heart infusion agar and poured onto standard agar plate. A single colony of each *Vibrio* strain was streaked and patched onto milk agar plate and the plate was incubated overnight at 37°C. Following incubation, the plate was observed for a zone of clearance around the bacterial growth. Results are shown in Appendix Figure S2, with both *V. mimicus* strains producing very high levels of protease activity on milk agar plate comparable to the protease activity elicited by *V. cholerae* O1 strain E7946. In contrast, *V. cholerae* O1 strains N16961 and O395 exhibited no protease activity in our experimental setting, consistent with an earlier report (*4*).

Quantitative protease assay. Quantitative protease assay was performed as described previously (*5*). Briefly, to determine quantitative protease activity, a single colony of *Vibrio* strain grown overnight on LB agar plate was inoculated in 3 ml brain heart infusion (BHI) broth and the culture was grown overnight at 37°C in a shaking incubator with a speed of 200 rpm. Next day, 0.5 ml of bacterial culture was inoculated in 500 ml BHI broth and the culture was grown overnight at 37°C in a shaking incubator (200 rpm). The culture was spun down by centrifugation at 12000 rpm for 40 min; the supernatant was carefully collected into another container and filter sterilized using 0.45 µm filter. The supernatant was used for protease activity.

To measure the protease activity, 0.65% casein solution was used as a substrate. Test tubes containing the casein solution was placed in a water bath at 37°C for 5 minutes for calibration purposes. Several dilutions of preprocessed supernatant from a desired test strain were added to the tubes, mixed by swirling, and incubated at a 37°C incubator for 10 minutes. After incubation, 110 mM Trichloroacetic acid (TCA) solution were added to each tube. Next, equal volume of protease from bovine pancreas were added to each tube and incubated for 30 minutes in a 37°C incubator. After the incubation, all the sample solutions and the blank were filter sterilized using syringe filters with 0.45µm pore size. Two ml of the filter sterilized sample solution and the blank from each tube were transferred to clean separate tubes where 5 ml of sodium carbonate solution and 1 ml of Folin's reagent were added. All the contents of the tubes were mixed by swirling and incubated at a 37°C incubator for 30 minutes. To compare the protease activity of the test samples, tyrosine standard solution with several dilutions were prepared by mixing water and tyrosine standard stock solution. 1.1 mM tyrosine standard stock solution at different volumes: 50 µl, 100 µl, 200 µl, 400 µl, 500 µl were inoculated into 5 separate test tubes and water was added in each of these tubes to make a total volume of 2 ml. A separate test tube containing no tyrosine standard stock solution but only 2 ml of water was considered as the blank. Similar to the tubes of the test samples and test blank, sodium carbonate solution (5 ml) and Folin's reagent (1ml) were added in all the tubes containing tyrosine standard solution and the standard blank. Similar to test samples and test blank, tubes containing standard and standard blank were filter sterilized using filters of 0.45µm pore size. Absorbance of all the test samples, test blank, standard samples and standard blank were measured at 660 nm using a spectrophotometer. Protease activity in a test sample was calculated and compared with the standard curve to quantify protease activity in that sample.

E3_US_2019 and D461B_US_2019 were found to produce similar level of proteolytic activities (51.72 and 52.8, respectively) indicating that both *V. mimicus* strains produced high level of protease that may have promoted severe diarrheal disease in patients from whom the strains were isolated.

Motility assay. Motility assays of *Vibrio* strains were performed as described previously (*6*). Briefly, A single colony of *Vibrio* strain of interest was streaked on LB agar and the plate was incubated overnight at 37°C incubator. Next, using a sterile needle the culture was inoculated into a motility agar plate (0.3% agar) and the plate was incubated at 37°C for 6 hours;

the plate was then observed with naked eyes to see if a strain migrated around the inoculating site reflecting motility phenomenon.

As shown in Appendix Figure 3A, while *V. cholerae* N16961S (smooth) variant produced motility with a diameter of 1.3 cm around the inoculating site, the motility of N16961R (rugose) and *V. mimicus* strains D461B_US_2019 and E3_US_2019 showed motility with diameter of 0.4, 0.8 and 0.5 cm, respectively. We previously reported that *V. cholerae* N16961R produces much less motility compared to its isogenic N16961S variant as the former produces copious amount of exopolysaccharide potentially inhibiting full potential of rugose variant's motility on motility agar plate (6). As expected, *V. cholerae flrA* mutant defective in flagella production exhibited no motility on the motility agar plate.

Biofilm assays. Quantitative assessment of biofilm produced by *Vibrio* strains was measured as described previously (*7*). Briefly, twenty-four well polystyrene plastic plates (Corning Incorporated, Corning, NY) were used as the surface for bacterial attachment. *Vibrio* strains, including *V. cholerae* O1 N16961S (smooth) and N16961R (rugose), N16961SΔ*vpsA* and two *V. mimicus* strains (E3_USA_2019 and D461B_US_2019) were included in the biofilm assay. The microorganisms were grown in L-broth and incubated overnight statically at room temperature. Following overnight incubation the cultures were discarded, and the tubes were then rinsed vigorously with distilled water to remove non-adherent cells, filled with 600 µl of a 0.1% crystal violet solution (Sigma, St. Louis, MO), allowed to incubate for 30 min at room temperature, and the tubes were again rinsed vigorously with water. Quantitative biofilm formation was determined by measuring the optical density at 570 nm (OD 570) of a solution produced by extracting cell-associated dye with 600 µl of dimethyl sulfoxide (DMSO) (Sigma, St. Louis, MO).

Data presented in Appendix Figure 3B show that both *V. mimicus* strains produced ~50% more biofilm than N16961S variant while they produced ~40% less biofilm than the N16961R strain. As expected, *V. cholerae* O395 and a N16961Δ*vpsA* mutant showed no or little biofilm formation as reported earlier (*7*). Taken together our data indicate that tested *V. mimicus* strains produce biofilm that may have contributed to the severe diarrhea seen in patients from whom we derived the strains.

Antimicrobial assay: Antimicrobial susceptibility test (AST) was performed for each strain against antimicrobial agents using disc diffusion method (*8*) by following the recommended guidelines of Clinical and Laboratory Standards Institute (CLSI, formally the National Committee for Clinical Laboratory Standards, NCLLS) as previously described (*9*,*10*).

Whole genome sequencing. DNA of the two *V. mimicus* strains was isolated and purified as follows: a single colony of each strain was grown in LB broth and the culture was incubated overnight at 37°C in a shaking incubator (250 rpm). After incubation, the culture was spun down by centrifugation; the supernatant was carefully decanted and the pellet was used for DNA extraction. Genomic DNA (gDNA) extraction was carried out using the Qiagen DNeasy Blood and Tissue kit (Qiagen, Inc.). Sample library construction was performed using the Nextera XT DNA Library Preparation Kit (Illumina, San Diego, CA 92122). Whole-genome sequencing of the isolates was carried out with the Illumina MiSeq for 500 cycles (Illumina, San Diego, CA 92122). Adaptor and raw sequence reads were filtered by length and quality by using the program Trimmomatic (*11*). After the FASTQ files were trimmed, the software SPAdes was used for de novo assembly, resulting in contigs for each isolate (*12*). The program Mauve (*13*) was used to compare whole genome alignment/mapping between *V. mimicus* strains used in this study and different *Vibrio* strains obtained from NCBI database (*Vibrio mimicus*; MB451 and VM223 strains, *Vibrio cholerae* O1 Inaba N16961 & *Vibrio parahaemolyticus*). The comparative genome alignment and mapping was performed to identify the contig(s) containing pathogenic island, virulence genes and different secretion system in our sequenced *V. mimicus* strains. The reference genomes indicated above were downloaded from the NCBI using corresponding accession number. The genes/ORF(s) were verified with BlastP search against Genbank NR database. RAST tool kit were used to annotate the genomes of *V. mimicus* strains (*14*).

Genomic features of *V. mimicus* strains. To decipher general features and identify virulence determinants incorporated into the genomes of two *V. mimicus* strains (E3_US_2019 and D461B_US_2019), we de novo assembled the whole genome sequences (WGSs) each of the two strains using SPAdes (*12*); we then annotated each genome using Prokka and RAST programs (*15*). Data are presented in Appendix Tables 2 and 4. The GC content ratio (GC%) of E3_US_2019 and D461B_US_2019 was 46.29% and 46.34%, respectively indicating the high degree of genetic similarity between these two *V. mimicus* strains. Moreover, the GC content of

V. mimicus strains is closely related to *V. cholerae* O1 GC content [~48%] (*16–18*). The genomes of E3_US_2019 and D461b_US_2019 harbor 753 and 542 ORFs that appear to have no assigned functions in NCBI database (hypothetical/conserved hypothetical genes). Furthermore, it appears that the strains contain 360 and 357 genetic subsystems, defined as a group or set of proteins that collectively perform a biologic process or promote a structural complex.

Hemolysin, *ctx*, protease identified in *V. mimicus* genomes. Using NCBI Blast search, we identified a series of major virulence factors in the genomes of two *V. mimicus* strains. The identified potential virulence factors are listed in Appendix Table 2. Although a subset of *V. mimicus* strains have been reported to harbor *ctx* and *ctx*-related genes, we found no evidence of *ctx* genes or CTX prophage integration site in the genomes of our *V. mimicus* strains. This finding led us to further screen strains for other potential virulence factors in the genomes of the strains that enabled these strains to cause severe diarrhea in patients from whom the strains were isolated. Both *V. mimicus* strains (E3_US_2019 and D461B_US_2019) possess one copy each of heat-stable (ST), heat-labile (LT) and enterotoxigenic (*hlyA*) gene. Heat labile toxins, also known as pore forming toxin and cytolysin, detected in the two strains were identical (both on nucleotide and amino acid level) while exhibiting 98.1% similarity to a hypervirulent *V. mimicus* strain SCCF01 isolated from a catfish in China (*17*). Similarly, the heat stable (ST) toxins of the two *V. mimicus* strains are identical (both on nucleotide and amino acid level) while they are 99.5% identical to the gene found in *V. mimicus* strain SCCF01 (*17*). The genomes of both *V. mimicus* strains harbor a series of genes encoding various proteases, including, VMP **(**Vm-HA), prtV protease, two types of zinc metalloprotease, protease IV and htpX protease. Previous reports (*19*) suggested that *V. mimicus* VMP protease (also known as Vm-HA protease) attributed to the virulence of the pathogen. The VMP protease modulates the activity of *V. mimicus* hemolysin by limited/selective proteolysis of hemolysin, a major virulence factor of *V. mimicus* strain (*19*). Both *V. mimicus* strains (E3_US_2019 and D461B_US_2019) encoded an identical VMP protein which exhibited 99.5% and 99.0% identity (on an amino acid level) to *V. mimicus* strain MB-451(VII_000553) and VM223 (VMA_000854), respectively. Both of our *V. mimicus* strains also harbored identical genes encoding neuraminidase protein in their genomes; the neuraminidase protein found in our strains showed 98.0% identity (on amino acid level) to a *V. mimicus* strain SCCF01(VM_15685).

Other potential virulence determinant in *V. mimicus* strains. Appendix Table 2 also illustrated a series of other potential virulence factors that are found in the genomes of our *V. mimicus* strains. These factors include genes (*rbmA-D*) encoding exopolysaccharide and biofilm formation, and major secretin systems (type I, II, III, IV and VI) (*20*). The Type II secretion system is known to secrete, hemolysin, proteases, flagellar proteins among others in *V. cholerae* O1 strains (*6*). While type III and type VI secretion systems are known to secrete effector molecules to modulate host-pathogen interactions in *Vibrio* pathogens (*20*), the Type IV secretion system promotes pilus formation promoting bacterial adhesion to biotic and abiotic surfaces that promotes both biofilm formation and pathogenesis (*20*,*21*). In summary, while the genomes of *V. mimicus* strains (E3_US_2019 and D461B_US_2019) do not have *ctx* genes, they have a plethora of potential virulence genes/proteins that may have contributed collectively and cumulatively to occurrence of severe cholera-like diarrheal disease.

Phylogeny of *V. mimicus* strains. Our dataset was composed of 35 *V. mimicus* isolates, which included the two isolates from this outbreak and isolates from NCBI's SRA database (Appendix Table 5). The FASTQ files from NCBI's SRA database were trimmed using the program Trimmomatic (*11*). After the FASTQ files were trimmed, the software SPAdes was used for de novo assembly (*12*), resulting in contigs for each isolate. The contigs were then reordered using Mauve Contig Mover and the *V. mimicus* CAIM-602 strain (RefSeq assembly accession: GCF_000338875.1) as the reference sequence (*22*). Using the reordered contigs, annotation was performed with the program Prokka (*15*). With the Prokka annotated GFF files, a core genome was produced with Roary, a software for creating and analyzing the pangenome (*23*). The core genome was produced and subsequently used to extract SNPs via the program SNP-sites (*24*). By using the core-genome SNPs (cgSNPs), we created a maximum likelihood (ML) tree using the best-fitting nucleotide substitution model with the program IQ-TREE (*25*). Ultrafast bootstrap approximation (*26*) (1000 replicates) was applied in IQ-TREE to assess the robustness of the internal branches of the ML phylogeny (*25*). Using IQ-TREE, we were also able conduct a likelihood mapping analysis, which calculates and maps the likelihood of all possible sequence quartets using the best-fitting nucleotide substitution model (*25*) (*27*). Likelihood analysis showed presence of phylogenetic in the dataset. The ML phylogeny was manipulated in R using the package ggtree (*28*) for publishing purposes. To further examine the

diversity of the isolates, the genetic pairwise distance was performed and analyzed in MEGA (*29*).

To identify the relationship across *V. mimicus* isolates collected in Florida (designated as D461B_US_2019 and E3_US_2019) and previously published isolates obtained in the U.S., Europe, and Asia (Appendix Table 3), we obtained the core genome alignment that comprised 3,039 genes. The ML phylogeny highlights the similarity between the two isolates collected from diarrheal stool samples in UF Shands Hospital in Gainesville, Florida, differentiating only in 7 SNPs across the core genome. Their high genetic similarity is also corroborated by genetic pairwise distance, d = 0.0000423. The ML phylogeny indicates that D461B_US_2019 and E3_US_2019 isolates form a well-supported monophyletic clade (bootstrap value >90%) with an isolate collected from the UK in 2015. Although isolates from the U.S. tend to cluster together within the ML phylogeny, there is no specific geographic clustering and isolates from Asia and Europe are intermixed with the U.S. ones.

References

1. Kim H-J, Ryu J-O, Lee S-Y, Kim E-S, Kim H-Y. Multiplex PCR for detection of the Vibrio genus and five pathogenic Vibrio species with primer sets designed using comparative genomics. BMC Microbiol. 2015;15:239. PubMed https://doi.org/10.1186/s12866-015-0577-3

2. Fan Y, Li Z, Li Z, Li X, Sun H, Li J, et al. Nonhemolysis of epidemic El Tor biotype strains of *Vibrio cholerae* is related to multiple functional deficiencies of hemolysin A. Gut Pathog. 2019;11:38. PubMed https://doi.org/10.1186/s13099-019-0316-7

3. Wang G, Fan C, Wang H, Jia C, Li X, Yang J, et al. Type VI secretion system-associated FHA domain protein TagH regulates the hemolytic activity and virulence of Vibrio cholerae. Gut Microbes. 2022;14:2055440. PubMed https://doi.org/10.1080/19490976.2022.2055440

4. Son MS, Taylor RK. Genetic Screens and Biochemical Assays to Characterize Vibrio cholerae O1 Biotypes: Classical and El Tor. Current protocols in microbiology. 2011;22a(6a2):6a.2.1–6a.2.17.

5. Cupp-Enyard C. Sigma's Non-specific Protease Activity Assay - Casein as a Substrate. Journal of visualized experiments: JoVE. 2008(19).

6. Ali A, Johnson JA, Franco AA, Metzger DJ, Connell TD, Morris JGJ Jr, et al. Mutations in the extracellular protein secretion pathway genes (*eps*) interfere with rugose polysaccharide

production in and motility of *Vibrio cholerae.* Infect Immun. 2000;68:1967–74. PubMed https://doi.org/10.1128/IAI.68.4.1967-1974.2000

7. Jubair M, Atanasova KR, Rahman M, Klose KE, Yasmin M, Yilmaz O, et al. Vibrio cholerae persisted in microcosm for 700 days inhibits motility but promotes biofilm formation in nutrient-poor lake water microcosms. PLoS One. 2014;9:e92883. PubMed https://doi.org/10.1371/journal.pone.0092883

8. Hudzicki J. Kirby-Bauer Disk Diffussion Susceptibility Test Protocol. American Society for Microbiology. 2009:1–23.

9. Jorgensen JH, Hindler JF, Reller LB, Weinstein MP. New consensus guidelines from the Clinical and Laboratory Standards Institute for antimicrobial susceptibility testing of infrequently isolated or fastidious bacteria. Clin Infect Dis. 2007;44:280–6. PubMed https://doi.org/10.1086/510431

10. Kiehlbauch JA, Hannett GE, Salfinger M, Archinal W, Monserrat C, Carlyn C. Use of the National Committee for Clinical Laboratory Standards guidelines for disk diffusion susceptibility testing in New York state laboratories. J Clin Microbiol. 2000;38:3341–8. PubMed https://doi.org/10.1128/JCM.38.9.3341-3348.2000

11. Bolger AM, Lohse M, Usadel B. Trimmomatic: a flexible trimmer for Illumina sequence data. Bioinformatics. 2014;30:2114–20. PubMed https://doi.org/10.1093/bioinformatics/btu170

12. Bankevich A, Nurk S, Antipov D, Gurevich AA, Dvorkin M, Kulikov AS, et al. SPAdes: a new genome assembly algorithm and its applications to single-cell sequencing. Journal of computational biology: a journal of computational molecular cell biology. 2012;19(5):455–77.

13. Rissman AI, Mau B, Biehl BS, Darling AE, Glasner JD, Perna NT. Reordering contigs of draft genomes using the Mauve aligner. Bioinformatics. 2009;25:2071–3. PubMed https://doi.org/10.1093/bioinformatics/btp356

14. Brettin T, Davis JJ, Disz T, Edwards RA, Gerdes S, Olsen GJ, et al. RASTtk: a modular and extensible implementation of the RAST algorithm for building custom annotation pipelines and annotating batches of genomes. Sci Rep. 2015;5:8365. PubMed https://doi.org/10.1038/srep08365

15. Seemann T. Prokka: rapid prokaryotic genome annotation. Bioinformatics. 2014;30:2068–9. PubMed https://doi.org/10.1093/bioinformatics/btu153

16. Heidelberg JF, Eisen JA, Nelson WC, Clayton RA, Gwinn ML, Dodson RJ, et al. DNA sequence of both chromosomes of the cholera pathogen *Vibrio cholerae.* Nature. 2000;406:477–83. PubMed https://doi.org/10.1038/35020000

17. Yu Z, Wang E, Geng Y, Wang K, Chen D, Huang X, et al. Complete genome analysis of *Vibrio mimicus* strain SCCF01, a highly virulent isolate from the freshwater catfish. Virulence. 2020;11:23–31. PubMed https://doi.org/10.1080/21505594.2019.1702797

18. Baker-Austin C, Oliver JD, Alam M, Ali A, Waldor MK, Qadri F, et al. Vibrio spp. infections. Nat Rev Dis Primers. 2018;4:8. PubMed https://doi.org/10.1038/s41572-018-0005-8

19. Hasan NA, Grim CJ, Haley BJ, Chun J, Alam M, Taviani E, et al. Comparative genomics of clinical and environmental Vibrio mimicus. Proc Natl Acad Sci U S A. 2010;107:21134–9. PubMed https://doi.org/10.1073/pnas.1013825107

20. Green ER, Mecsas J. Bacterial Secretion Systems: An Overview. Microbiol Spectr. 2016;4:4.1.13. PubMed https://doi.org/10.1128/microbiolspec.VMBF-0012-2015

21. Pukatzki S, Ma AT, Revel AT, Sturtevant D, Mekalanos JJ. Type VI secretion system translocates a phage tail spike-like protein into target cells where it cross-links actin. Proc Natl Acad Sci U S A. 2007;104:15508–13. PubMed https://doi.org/10.1073/pnas.0706532104

22. Guardiola-Avila I, Acedo-Felix E, Noriega-Orozco L, Yepiz-Plascencia G, Sifuentes-Romero I, Gomez-Gil B. Draft Genome Sequence of Vibrio mimicus Strain CAIM 602T. Genome Announc. 2013;1:e0008413. PubMed https://doi.org/10.1128/genomeA.00084-13

23. Page AJ, Cummins CA, Hunt M, Wong VK, Reuter S, Holden MT, et al. Roary: rapid large-scale prokaryote pan genome analysis. Bioinformatics. 2015;31:3691–3. PubMed https://doi.org/10.1093/bioinformatics/btv421

24. Page AJ, Taylor B, Delaney AJ, Soares J, Seemann T, Keane JA, et al. *SNP-sites*: rapid efficient extraction of SNPs from multi-FASTA alignments. Microb Genom. 2016;2:e000056. PubMed https://doi.org/10.1099/mgen.0.000056

25. Nguyen LT, Schmidt HA, von Haeseler A, Minh BQ. IQ-TREE: a fast and effective stochastic algorithm for estimating maximum-likelihood phylogenies. Mol Biol Evol. 2015;32:268–74. PubMed https://doi.org/10.1093/molbev/msu300

26. Minh BQ, Nguyen MA, von Haeseler A. Ultrafast approximation for phylogenetic bootstrap. Mol Biol Evol. 2013;30:1188–95. PubMed https://doi.org/10.1093/molbev/mst024

27. Schmidt HA, Strimmer K, Vingron M, von Haeseler A. TREE-PUZZLE: maximum likelihood phylogenetic analysis using quartets and parallel computing. Bioinformatics. 2002;18:502–4. PubMed https://doi.org/10.1093/bioinformatics/18.3.502

28. Yu G, Smith DK, Zhu H, Guan Y, Lam TT-Y. ggtree: an r package for visualization and annotation of phylogenetic trees with their covariates and other associated data. Methods Ecol Evol. 2017;8:28–36. https://doi.org/10.1111/2041-210X.12628

29. Tamura K, Peterson D, Peterson N, Stecher G, Nei M, Kumar S. MEGA5: molecular evolutionary genetics analysis using maximum likelihood, evolutionary distance, and maximum parsimony methods. Mol Biol Evol. 2011;28:2731–9. PubMed https://doi.org/10.1093/molbev/msr121

30. Miyoshi S, Sasahara K, Akamatsu S, Rahman MM, Katsu T, Tomochika K, et al. Purification and characterization of a hemolysin produced by Vibrio mimicus. Infect Immun. 1997;65:1830–5. PubMed https://doi.org/10.1128/iai.65.5.1830-1835.1997

31. Mizuno T, Nanko A, Maehara Y, Shinoda S, Miyoshi S. A novel extracellular protease of Vibrio mimicus that mediates maturation of an endogenous hemolysin. Microbiol Immunol. 2014;58:503–12. PubMed https://doi.org/10.1111/1348-0421.12177

32. Chowdhury MA, Miyoshi S, Shinoda S. Purification and characterization of a protease produced by Vibrio mimicus. Infect Immun. 1990;58:4159–62. PubMed https://doi.org/10.1128/iai.58.12.4159-4162.1990

33. Vaitkevicius K, Lindmark B, Ou G, Song T, Toma C, Iwanaga M, et al. A Vibrio cholerae protease needed for killing of Caenorhabditis elegans has a role in protection from natural predator grazing. Proc Natl Acad Sci U S A. 2006;103:9280–5. PubMed https://doi.org/10.1073/pnas.0601754103

34. Engel LS, Hill JM, Caballero AR, Green LC, O'Callaghan RJ. Protease IV, a unique extracellular protease and virulence factor from Pseudomonas aeruginosa. J Biol Chem. 1998;273:16792–7. PubMed https://doi.org/10.1074/jbc.273.27.16792

35. Wang Q, Wang H, Jiang Y, Lv M, Wang X, Chen L. Biotransformation mechanism of Vibrio diabolicus to sulfamethoxazole at transcriptional level. J Hazard Mater. 2021;411:125023. PubMed https://doi.org/10.1016/j.jhazmat.2020.125023

36. Huang HH, Lin YT, Chen PY, Li LH, Ning HC, Yang TC. ClpA and HtpX Proteases Are Involved in Intrinsic Aminoglycoside Resistance of Stenotrophomonas maltophilia and Are Potential

Aminoglycoside Adjuvant Targets. Antimicrob Agents Chemother. 2018;62:e00554-18. PubMed https://doi.org/10.1128/AAC.00554-18

37. Jermyn WS, Boyd EF. Characterization of a novel Vibrio pathogenicity island (VPI-2) encoding neuraminidase (nanH) among toxigenic Vibrio cholerae isolates. Microbiology (Reading). 2002;148:3681–93. PubMed https://doi.org/10.1099/00221287-148-11-3681

38. Thomas S, Holland IB, Schmitt L. The Type 1 secretion pathway - the hemolysin system and beyond. Biochim Biophys Acta. 2014;1843:1629–41. PubMed https://doi.org/10.1016/j.bbamcr.2013.09.017

39. Korotkov KV, Johnson TL, Jobling MG, Pruneda J, Pardon E, Héroux A, et al. Structural and functional studies on the interaction of GspC and GspD in the type II secretion system. PLoS Pathog. 2011;7:e1002228. PubMed https://doi.org/10.1371/journal.ppat.1002228

40. Jarvis KG, Girón JA, Jerse AE, McDaniel TK, Donnenberg MS, Kaper JB. Enteropathogenic Escherichia coli contains a putative type III secretion system necessary for the export of proteins involved in attaching and effacing lesion formation. Proc Natl Acad Sci U S A. 1995;92:7996–8000. PubMed https://doi.org/10.1073/pnas.92.17.7996

41. Park KS, Ono T, Rokuda M, Jang MH, Okada K, Iida T, et al. Functional characterization of two type III secretion systems of Vibrio parahaemolyticus. Infect Immun. 2004;72:6659–65. PubMed https://doi.org/10.1128/IAI.72.11.6659-6665.2004

42. Matz C, Nouri B, McCarter L, Martinez-Urtaza J. Acquired type III secretion system determines environmental fitness of epidemic Vibrio parahaemolyticus in the interaction with bacterivorous protists. PLoS One. 2011;6:e20275. PubMed https://doi.org/10.1371/journal.pone.0020275

43. Borgeaud S, Metzger LC, Scrignari T, Blokesch M. The type VI secretion system of Vibrio cholerae fosters horizontal gene transfer. Science. 2015;347:63–7. PubMed https://doi.org/10.1126/science.1260064

44. Joshi A, Kostiuk B, Rogers A, Teschler J, Pukatzki S, Yildiz FH. Rules of Engagement: The Type VI Secretion System in Vibrio cholerae. Trends Microbiol. 2017;25:267–79. PubMed https://doi.org/10.1016/j.tim.2016.12.003

45. Henderson DP, Payne SM. Characterization of the Vibrio cholerae outer membrane heme transport protein HutA: sequence of the gene, regulation of expression, and homology to the family of TonB-dependent proteins. J Bacteriol. 1994;176:3269–77. PubMed https://doi.org/10.1128/jb.176.11.3269-3277.1994

46. Payne SM, Mey AR, Wyckoff EE. Vibrio Iron Transport: Evolutionary Adaptation to Life in Multiple Environments. Microbiol Mol Biol Rev. 2015;80:69–90. PubMed https://doi.org/10.1128/MMBR.00046-15

47. Wyckoff EE, Valle AM, Smith SL, Payne SM. A multifunctional ATP-binding cassette transporter system from Vibrio cholerae transports vibriobactin and enterobactin. J Bacteriol. 1999;181:7588–96. PubMed https://doi.org/10.1128/JB.181.24.7588-7596.1999

48. Kuehl CJ, Crosa JH. The TonB energy transduction systems in Vibrio species. Future Microbiol. 2010;5:1403–12. PubMed https://doi.org/10.2217/fmb.10.90

49. Sakib SN, Reddi G, Almagro-Moreno S. Environmental role of pathogenic traits in *Vibrio cholerae.* J Bacteriol. 2018;200:e00795–17. PubMed https://doi.org/10.1128/JB.00795-17

50. Shapiro BJ, Levade I, Kovacikova G, Taylor RK, Almagro-Moreno S. Origins of pandemic Vibrio cholerae from environmental gene pools. Nat Microbiol. 2016;2:16240. PubMed https://doi.org/10.1038/nmicrobiol.2016.240

51. Merrell DS, Bailey C, Kaper JB, Camilli A. The ToxR-mediated organic acid tolerance response of Vibrio cholerae requires OmpU. J Bacteriol. 2001;183:2746–54. PubMed https://doi.org/10.1128/JB.183.9.2746-2754.2001

52. Manning PA. The *tcp* gene cluster of *Vibrio cholerae.* Gene. 1997;192:63–70. PubMed https://doi.org/10.1016/S0378-1119(97)00036-X

53. McCarter LL. Polar flagellar motility of the Vibrionaceae. [table of contents.]. Microbiol Mol Biol Rev. 2001;65:445–62. PubMed https://doi.org/10.1128/MMBR.65.3.445-462.2001

54. Echazarreta MA, Kepple JL, Yen LH, Chen Y, Klose KE. A Critical Region in the FlaA Flagellin Facilitates Filament Formation of the Vibrio cholerae Flagellum. J Bacteriol. 2018;200:e00029-18. PubMed https://doi.org/10.1128/JB.00029-18

55. Ran Kim Y, Haeng Rhee J. Flagellar basal body flg operon as a virulence determinant of Vibrio vulnificus. Biochem Biophys Res Commun. 2003;304:405–10. PubMed https://doi.org/10.1016/S0006-291X(03)00613-2

56. Butler SM, Camilli A. Both chemotaxis and net motility greatly influence the infectivity of Vibrio cholerae. Proc Natl Acad Sci U S A. 2004;101:5018–23. PubMed https://doi.org/10.1073/pnas.0308052101

57. Fong JC, Yildiz FH. The rbmBCDEF gene cluster modulates development of rugose colony morphology and biofilm formation in Vibrio cholerae. J Bacteriol. 2007;189:2319–30. PubMed https://doi.org/10.1128/JB.01569-06

58. Güvener ZT, McCarter LL. Multiple regulators control capsular polysaccharide production in Vibrio parahaemolyticus. J Bacteriol. 2003;185:5431–41. PubMed https://doi.org/10.1128/JB.185.18.5431-5441.2003

59. Sau S, Bhasin N, Wann ER, Lee JC, Foster TJ, Lee CY. The Staphylococcus aureus allelic genetic loci for serotype 5 and 8 capsule expression contain the type-specific genes flanked by common genes. Microbiology (Reading). 1997;143:2395–405. PubMed https://doi.org/10.1099/00221287-143-7-2395

60. Ali A, Rashid MH, Karaolis DKR. High-frequency rugose exopolysaccharide production by *Vibrio cholerae.* Appl Environ Microbiol. 2002;68:5773–8. PubMed https://doi.org/10.1128/AEM.68.11.5773-5778.2002

61. Sinha-Ray S, Ali A. Mutation in *flrA* and *mshA* Genes of *Vibrio cholerae* Inversely Involved in *vps*-Independent Biofilm Driving Bacterium Toward Nutrients in Lake Water. Front Microbiol. 2017;8:1770. PubMed https://doi.org/10.3389/fmicb.2017.01770

Appendix Table 1. Antibiotic susceptibility tests for *V. mimicus* strains using a battery of commonly used antibiotics.

Antimicrobial agent (Amount in µg)	Antibiotic susceptibility test (S/R/I)*	
	E3_US_2019	D461B_US_2019
Doxycycline (30)	S	S
Ceftriaxone (30)	S	S
Ampicillin (10)	S	I
Chloramphenicol (30)	S	S
Azithromycin (15)	S	S
Cephalothin (30)	S	I
Gentamicin (120)	S	S
Amikacin (30)	S	S
Sulphonamide (1000)	R	R
Cefixime (5)	S	S
Ciprofloxacin (5)	S	S
Sulfasoxazole (1000)	S	S
Cefotaxime (30)	S	S
Erythromycin (15)	I	I
Ceftazidime (30)	S	S
Cotrimoxazole (25)	S	S
Streptomycin (10)	R	R
Nalidixic acid (30)	S	S
Tetracycline (30)	S	S

*S, R, and I represent susceptible, resistance, and intermediate, respectively for each antibiotic used.

Appendix Table 2. Identification of major virulence factors detected in the genomes of *V. mimicus* strains D461B_US_2019 and E3_US_2019

Virulence category/type	Virulence factor(s)	Potential virulence gene(s)/ protein(s)	Known/putative function(s)	References
Toxin	Hemolysins and enterotoxin	*Vibrio mimicus* Hemolysin (VMH), heat stable hemolysin and enterotxigenic HlyA	Primarily involved in hemolytic activity of erythrocyte. VMH, cytolysin, create transmembrane pore in erythrocyte and disrupts the cell membrane. HlyA has enterotoxigenic activity.	(*30*) (*19*)
	Proteases	*Vibrio mimicus* protease (VMP) metalloproteases, PrtV protease, protease IV, HtpX protease and neuraminidase (sialidase).	VMP modulate the activity of *V. mimicus* hemolysin (Pro-VMH) and help activating the mature toxin; plays role in fluid accumulation in rabbit illeal loops; PrtV protease is necessary to kill *Caenorhabditis elegans;* protease IV degrades biomolecules related to immune responses to infection; HtpX protease degrades defective protein in gram-negative bacteria and up-regulates in response to the exposure to kanamycin; neuraminidase metabolize sialic acid.	(*19*) (*31*) (*32*) (*33*) (*34*) (*35*) (*36*) (*37*)
Secretion system	Type I secretion system	RTX	Secretion of HlyA from the cytosol to extracellular milieu.	(*38*)
	Type II secretion system	*gspC* – *gspN* (12 genes)	Secrete a broad range of proteins, including hemolysin, proteases, and exopolysaccharide from the periplasm to the external milieu.	(*39*)
	Type III secretion system	*toxR*, *vcsQ*, *vcsU2*, *vcsV2*, *vopM/VsaC*, *espD*, *vcsJ2*, *vcsS2, vcsC2/SctC*, *vttR/toxR*, *vcsT2*, *vopA*, *vopB2*, *vopC*, *vopD2*, *vopF*, *vopH*, and *vopL*	Contribute primarily to cytotoxicity, required for intestinal colonization and intestinal fluid accumulation in rabbit illeal loop, involved in secretion and translocation of virulence factor or effector proteins into eukaryotic cell and promotes persistence during interaction with predatory protists.	(*40*) (*41*) (*42*)
	Type VI secretion system	*hcP*-1, *hcP*-2, *vasX*, *vgrG*-1–3, *vasA* - *vasL* and *vipA*-*vipB*	T6SS involves in variety of functions, including virulence to eukaryotic and prokaryotic host, antibacterial activity, confer advantage during competition with other bacteria, foster horizontal gene transfer and participate in metal ion uptake such as zinc, manganese, and iron.	(*43*) (*44*)
Iron uptake	Heme receptor	HutA	Outer membrane receptor that recognizes and transports heme iron.	(*45*)
	Siderophore receptor	FhuA	Assist in ferrichrome uptake.	(*46*)
	Siderophores (Enterobactin, Vibriobactin, and Aerobactin)	*vctA, irgA, viuA*, *viuB* and *iutA*	Genes express during iron starvation, for example, ViuABCD or VctABCD transporters promote transportation of iron-vibriobactin siderophore complex.	(*46*) (*47*)
	TonB1 system	ExbB1, ExbD1 and TonB1	Plays crucial role in iron and vitamin B12 transport; requires for energy transduction.	(*46*) (*47*) (*48*)
	TonB2 system	ExbB2, ExbD2 and TonB2	Assists in iron transport.	(*46*)
	Transport of iron complexes	*hmuV*, *hutC*, *fhuB-fhuD*, *vctC*, *vctD*, *vctG* and *vctP*	Heme iron receptors serve as siderophore transporters.	(*46*)
	Feo system	FeoA-FeoB	Act as a major route for ferrous iron transport.	(*46*)
	Fbp system	FbpA-FbpC	Promote ferric iron transport.	(*46*)
Adherence	Outer membrane protein	*ompU*	Contribute to intestinal colonization, biofilm formation and confer resistance to bile, organic acid, and antibiotic polymyxin B.	(*21*) (*22*) (23) (*49*) (*50*) (*51*)
	Mannose-sensitive hemagglutinin	*mshA-mshQ*	Promote adherence, putative role in attachment to plankton, biofilm formation and serve as phage receptor.	(*19*)

Virulence category/type	Virulence factor(s)	Potential virulence gene(s)/ protein(s)	Known/putative function(s)	References
	Type IV pilus	*pilA-pilD*	Promote colonization in the host gut, and initiate biofilm formation on the biotic and abiotic surfaces in the environment.	(*52*)
Flagellar system	Flagella biosynthesis and motility<br>Flagellar motor protein	*flaA*, *flaC-E*, *flab*, *flag*, *flhB*, *flgA*, *flgB*, *flgM-P*, *flgT*, *flgA*-*flgN* and *fliE-fliR*<br>*motA*, *motB*, *motX* and *motY*	Flagella biosynthesis and motility. Swarming motility, production of conditional lateral and polar flagella.<br>Rotation of flagella in any orientation as and when required	(*53*) (*54*) (*55*)
Chemotaxis	Chemotaxis protein	*cheA*, *cheB*, *cheR*, *cheV*, *cheW*,*cheY* and *cheZ*	Play critical roles during entry to the host; site-specific colonization and infection in host intestine.	(*56*)
Poly-saccharide	Exopoly-saccharide	*rbmA-D*	Genes involved in the production of exopolysaccharide promoting biofilm formation.	(*57*)
	Capsular poly-saccharide	CpsA-D; Cap5L and two capsular polysaccharide biosynthesis protein	Required for capsular polysaccharide formation, resist antibiotics' actions	(*58*) (*59*)

Appendix Table 3. Bacterial strains used in this study

Strain	Description	Reference
V. cholerae strains		
N16961S	A wild-type, smooth, O1 El Tor strain isolated in Bangladesh in 1971	(*60*)
N16961R	A rugose variant of N16961S strain	(*60*)
E7946	O1 El Tor, ogawa clinical toxigenic isolate in 1978 from Bahrain	Laboratory Collection
O395	O1 classical, ogawa clinical toxigenic isolate in 1965 from India	Laboratory Collection
AAS84 (N16961SΔ*flrA*)	A 49-bp *flrA* internal deletion mutant created in the background of N16961S strain	(*61*)
AA218 (N16961SΔ*vpsA*)	A Δ*vpsA* in-frame null mutation created in the background of N16961S strain	(*7*)
V. mimicus strains		
E3_US_2019	Strain isolated from a patient with acute diarrhea admitted in UFHealth/Shands Hospital in Gainesville, Florida	This study
D461B_US_2019	Strain isolated from a patient with acute diarrhea admitted in UFHealth/Shands Hospital in Gainesville, Florida	This study

Appendix Table 4. Comparison of general features between two clinical *V. mimicus* strains

Genomic feature	*V. mimicus* E3_US_2019	*V. mimicus* D461B_US_2019
Genome size (Mb)	4.63	4.47
GC content (%)	46.29	46.34
ORFs encoding functional protein	3463	3438
ORFs with no assigned function	753	542
Number of subsystem present	360	357

Appendix Table 5. List of *V. mimicus* strains used for phylogenetic analysis in this study

Taxa	Strain name	Origin	Collection Date	GenBank accession number
218344_UK_2015	218344	UK	2015	SRR7637794
11435_US_1977	NCTC11435	USA_NC	1977	GCF_900460385.1
2011V-1073	2011V-1073	USA	unknown	GCF_009665195.1
269_80_US_1980	ATCC 33654	USA_LA	1975/1980	GCF_008464965.1
523_80_US_1980	523–80	USA	1980	GCF_000736955.1
ARGOS_112_US	FDAARGOS_112	USA_LA	unknown	GCF_001558475.2
ARGOS_113_US	FDAARGOS_113	USA_TN	unknown	GCF_001471395.2
CAIM_1882_MX_2013	CAIM_1882_MX_2013	Mexico, Guaymas	9/24/2012	GCF_000473785.1
CAIM_1883_MX_2012	CAIM_1883_MX_2012	Mexico, Guaymas	9/24/2012	GCF_000473825.1
CAIM_602_US_1981	CAIM_602_US_1981	USA_NC	1981	GCF_000338875.1
D461B_US_2019	D461B	USA_FL	6/19/2019	Submitted
E3_US_2019	E3	USA_FL	6/19/2019	Submitted
MB_451_BD	MB_451_BD	Bangladesh	unknown	GCF_000176375.1
OSF090171_US_2018	CFSAN090171	USA_WA	2018	SRR8535051
OSF090194_US_2018	CFSAN090194	USA_WA	2018	SRR8535197
PN155_US_2016	PNUSAV000155	USA_WY	2016	SRR6359167
SCCF01_CN_2013	SCCF01	China: Sichuan	8/26/2013	GCF_001767355.1
SX_4_CN_2009	SX-4	China	2009	GCF_000222145.1
VM223_BR_2009	VM223	Brazil, Sao Paulo	unknown	GCF_000176415.1
VM573_US_1990	VM573	USA	1990	GCF_000175995.1
VM603_BR_1990	VM603_BR_1990	Brazil. Amazon	1990	GCF_000175975.1
WAPHL_US_2017	WAPHLVBRA00025	U.S., WA	2017–10	SRR6278408
F9458	F9458	USA	unknown	NZ_CP046846.1, NZ_CP046847.1
MCCC1A02602	MCCC1A02602	China	2020–08	GCF_019846155.1
MCCC1H00078	MCCC1H00078	China	unknown	GCF_019846815.1
N2733	N2733	unknown (China?)	unknown	GCF_008084745.1
N2763	N2763	unknown (China?)	unknown	GCF_008084405.1
N2781	N2781	unknown (China?)	unknown	GCF_008083965.1
N2789	N2789	unknown (China?)	unknown	GCF_008083745.1
N2790	N2790	unknown (China?)	unknown	GCF_008083775.1
N2810	N2810	unknown (China?)	unknown	GCF_008083535.1
N2816	N2816	unknown (China?)	unknown	GCF_008083465.1
06_2455	06–2455	USA_PA	8/10/2006	NZ_CP046823.1, NZ_CP046824.1
07_2442	07–2442	USA_PA	8/10/2006	NZ_CP046825.1, NZ_CP046826.1
08_2414	08–2414	USA_PA	8/10/2006	GCF_014525095.1

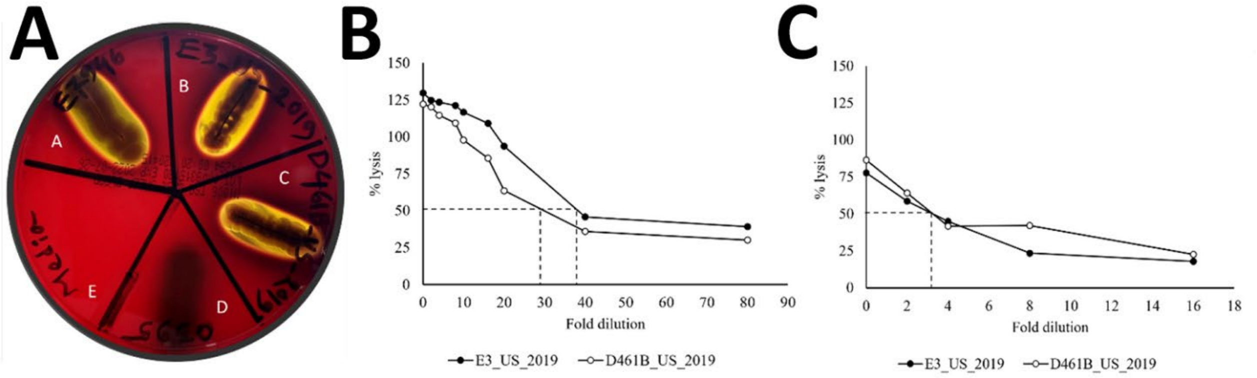

Appendix Figure 1. Hemolysin Assays. A) Qualitative hemolysis assay of *Vibrio* strains performed on 5% sheep blood agar plate incubated overnight at 37°C incubator. The strains exhibited hemolytic activity showed a clear zone of hemolysis around their respective spot as observed by naked eyes. In contrast, strain(s) lacking hemolytic activity or spot not inoculated with microorganism exhibited no zone of clearance. A, *V. cholerae* O1 strain E7946; B, *V. mimicus* strain E3_USA_2019; C, *V. mimicus* strain D461B_US_2019; D, *V. cholerae* O1 strain O395; E, media control with no microorganism was cultured on the plate. B). Quantitative hemolytic assay of *V. mimicus* strains. The hemolytic activity of the cell-free supernatant of *V. mimicus* strains, including the strains E3_USA_2019 and D461B_US_2019 grown in Syncase medium was compared to the complete lysis (100%) of erythrocytes induced by the exposure of erythrocytes to the Triton-X 100. The x-axis indicates fold dilution of the cell-free supernatants while y-axis indicates % lysis of the erythrocytes resulting in from the release of hemoglobin. The filled circle indicates the hemolytic activity of the test strain E3_USA_2019 while the open circle shows the activity of the strain D461B_US_2019. C) Quantitative hemolytic assay of *V. mimicus* strains associated with cytoplasmic extract. The hemolytic activity associated with the cytoplasmic extracts of *V. mimicus* strains, including the strains E3_US_2019 and D461B_US_2019 grown in Syncase medium was compared to the complete lysis (100%) of erythrocytes induced by the exposure of erythrocytes to the Triton-X 100. The x-axis indicates fold dilutions of the cytoplasmic extract contents while the y-axis reflects % lysis of the erythrocytes resulting in from the release of hemoglobin. The filled circle indicates the hemolytic activity of the test strain E3_USA_2019 while the open circle shows the activity of the strain D461B_US_2019. Both E3_USA_2019 and D461B_US_2019 strains displayed 50% hemolytic activity at a fold dilution of ~3.3 by dashed lines. The amount of hemolytic toxin necessary to elicit 50% release of hemoglobin or to cause 50% hemolysis was defined as the hemolysin unit (HU_{50}).

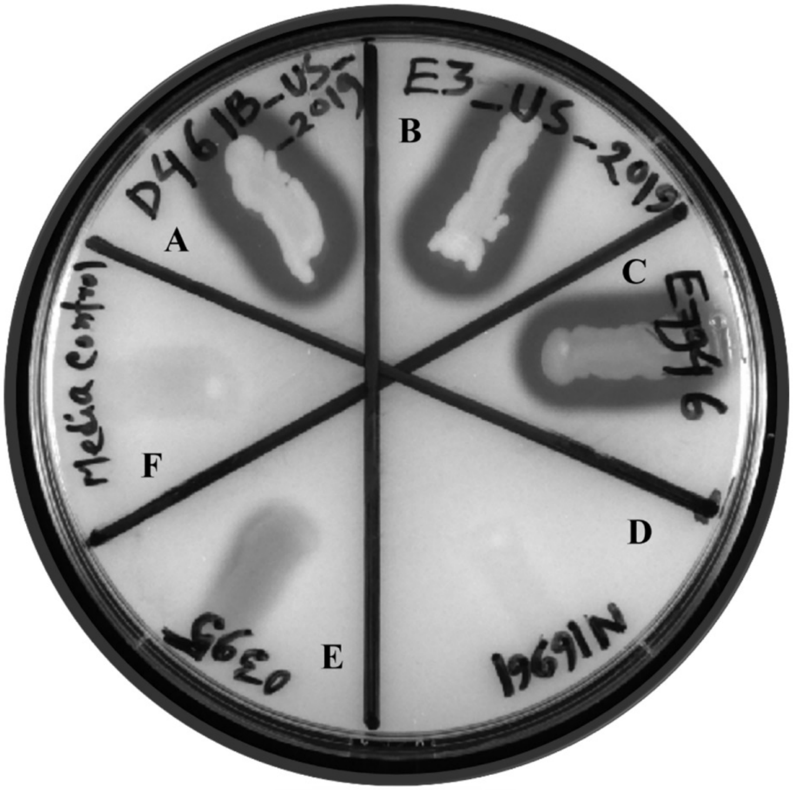

Appendix Figure 2. Qualitative protease activity of *Vibrio* strains performed on milk agar plate incubated overnight at 37°C incubator. The strains exhibited proteolytic activity showed a zone of clearance around their respective spot on the agar plate as observed by naked eyes. In contrast strain(s) lacking proteolytic activity or spot not inoculated with microorganism exhibited no zone of clearance. A, media control with no microorganism was spotted on the plate; B, *V. mimicus* strain D461B_US_2019; C, *V. mimicus* strain E3_US_2019; D, *V. cholerae* O1 strain E7946; E, *V. cholerae* O1 strain N16961; F, *V. cholerae* O1 strain O395.

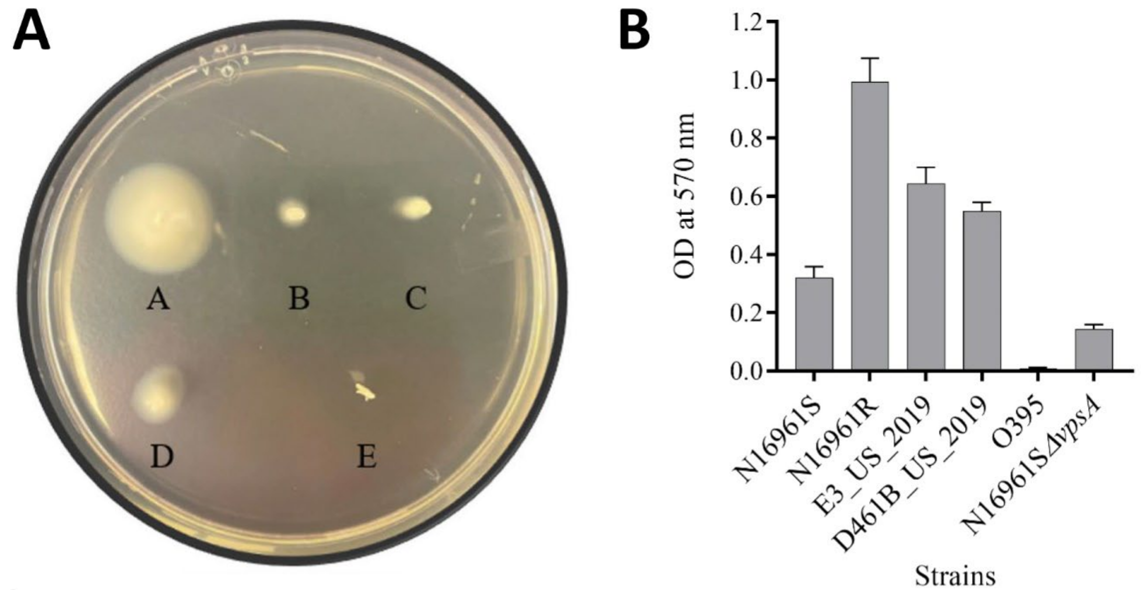

Appendix Figure 3. A) Motility assay of *Vibrio* strains. The motility assay was performed on motility agar as described previously (*7*) and swarming behavior of the tested strains was measured by their ability to move from inoculating site (*7*). A, *V. cholerae* O1 strain N16961S (smooth); B, *V. cholerae* O1 strain N16961R (rugose); C, *V. mimicus* strain E3_USA_2019; D, *V. mimicus* strain D461B_US_2019; E, *V. cholerae* N16961Δ*flaA*. B) Biofilm assay of *Vibrio* strains. The standard biofilm assay was performed as described previously. The data represent the average of 6 independent sets of experiment. The microorganism included in the assay included: *V. cholerae* O1 strain N16961S (smooth) and N16961R (rugose) variants, *V. cholerae* N16961SΔ*vpsA*, *V. cholerae* O1 O395 strain and *V. mimicus* strains, including E3_USA_2019 and D461B_US_2019.
